# Patients’ preferences for telemedicine versus in-clinic consultation in primary care during the COVID-19 pandemic

**DOI:** 10.1186/s12875-022-01640-y

**Published:** 2022-02-22

**Authors:** I. Mozes, D. Mossinson, H. Schilder, D. Dvir, O. Baron-Epel, A. Heymann

**Affiliations:** 1 School of Public Health, Haifa University, 199 Aba Khoushy Ave. Mount Carmel, 3498838 Haifa, Israel; 2 Meuhedet Health Services, 124 Ibn Gabirol, 6203854 Tel Aviv, Israel; 3 Medical School, Tel Aviv University, 69978 Ramat Aviv, Israel

**Keywords:** Telemedicine, Hybrid primary care, Discrete choice experiment, Patients’ preferences, Covid-19

## Abstract

**Background:**

The Hybrid Patient Care system integrates telehealth and in-clinic consultation. While COVID-19 increased telehealth use, healthcare providers are still seeking the best combination of virtual and in-clinic consultation. Understanding patients’ tele-consultation-related preferences is vital for achieving optimal implementation. The discrete choice experiment (DCE) is the stated preference technique for eliciting individual preferences and is increasingly being used in health-related applications.

The study purpose was to evaluate attributes and levels of the DCE regarding patients’ preferences for telemedicine versus traditional, in-clinic consultation in primary care during the COVID-19 pandemic, in order to facilitate successful implementation.

**Methods:**

A three-phase structure was used in the qualitative stage of the DCE: (1) a literature review and preparation of interview guides; (2) Eight focus group interviews comprised of 26 patients and 33 physicians; and (3) Attribute selection: a ranking exercise among 48 patients. The Think Aloud technique, in which respondents are asked to verbalize their thoughts, was used in the focus groups. Interview data were analyzed by thematic analysis.

**Results:**

Eight attributes were proposed by the patients in the focus groups. The four most important attributes were then selected in pre-testing, and are described in this study: Availability, time until the appointment, severity of the medical problem, patient-physician relationship, and flexible reception hours.

**Conclusions:**

This study has a theoretical contribution in post-COVID-19 patients’ preferences in Hybrid Medicine patient care. This provides a foundation to assess the rigors of this stage and provide additional evidence to the limited existing literature on attributes development for DCE patient preferences.

**Supplementary Information:**

The online version contains supplementary material available at 10.1186/s12875-022-01640-y.

## Background

*Telemedicine*, is conceived of as an integrated system of healthcare delivery that employs telecommunications and computer technology as a substitute for face-to-face contact between physician and patient [1]. Telemedicine offers several advantages including increased care accessibility [2, 3], decreased transportation barriers as well as costs [4, 5], and patient empowerment [6, 7]. The Hybrid Patient Care system integrates telehealth and in-person treatment. There are various methods to implement the use of one or more telehealth systems. This study focuses on the “Hybrid Medicine” (HM) system implemented in Israel, which enables the patient the option to receive, at no cost, health services from his/her primary care physician via one or more of the following channels:

(1) Face-to-face, traditional in-clinic consultation (FV); (2) Video Visits (VV) via the Web or a mobile application; (3) Phone Visits (PV); and (4) the Store-and-Forward written digital form (SFF) option.

During the recent COVID-19 pandemic, there was a rapid adoption of telemedicine consultation in primary care. This is because telemedicine visits prevent exposure to the virus and reduce the possibility of contracting it [8, 9]. Furthermore, at-risk patients, such as those suffering from diabetes or hypertension, prefer to reduce contact with their physician as well as with other patients (for example, regarding queuing time before the consultation). Therefore, during the pandemic, these patients preferred and made more use of telemedicine services [10].

Literature on patients’ preferences related to the use of telemedicine channels in the pre-COVID-19 period describes the different time and cost considerations, primary care practice (PCP) characteristics, relationship with PCPs, and perceived quality of consultation [11–14]. Patients’ preferences and considerations, however – when faced with a choice of one or more of several diverse telemedicine and HM channels such as video consultations and phone visits have yet to receive significant research attention. Store-and-Forward telemedicine is another such option. The Store& Forward digital form is a way for the patient to communicate with the PCP in an asynchronous manner, which allows patients to request a prescription for medication, send test results or other general requests. The existing literature focuses mainly on two options: face-to-face visits vs. video visits or video vs. phone visits [15–17].

Moreover, there are limited studies with validated attributes and levels among targeted populations; and in many studies, the working methods were insufficiently described and reported [18, 19]. In addition, the Think Aloud method, which has the potential to deepen our understanding of the attributes and levels that influence patients’ choice, has also received scant research attention [20]. Therefore, we investigate the concepts that could form the attributes that influence patients’ choice regarding HM, and which quantify patients’ preferences, leading to successful implementation during the COVID-19 period.

## Methods

### Study design and sample

Discrete Choice Methodology (DCE) was used, as this tool deepens our understanding of individuals’ preferences. This technique has become increasingly popular in recent health policy and economics research, due to its unique benefits in preferences elicitation [21–23].

*DCE* is a stated-preference approach that asks respondents to make hypothetical choices between options defined by a series of dimensions, each of which can take one out of a finite list of possible levels. The first qualitative stage has crucial importance in the success of DCE research and an accurate understanding of preferences. To identify key attributes and levels in this qualitative DCE stage, three steps are conducted: (1) a literature review, (2) a pre-experiment qualitative stage via focus groups with patients and PCPs, and (3) a ranking exercise.

During the literature screening researchers found data about variables important to patients and with this information, we formulated interview guides, understood what questions to ask. Attributes relating to patients’ preferences about primary care in general, and telemedicine in primary care in particular, were found and used by the researchers to formulate interview guides. In the second step, four focus groups comprised of 24 user-patients of hybrid primary care services and four groups comprised of 33 PCPs working in Hybrid Medicine were interviewed in order to collect qualitative data according to the Good Practices Guide [24]. The Think Aloud technique was used in the focus groups. The first author (IM) wrote a diary, filled out after each focus group and research meeting, thereby systematically sharing insights with the whole research group on an ongoing basis during the data collection phase. The study was approved by the Ethics Committee of the Meuhedet HMO, where the study took place. The methods and the results are reported according to the Consolidated Criteria for Reporting Qualitative Research (COREQ) guidelines [25].

The current study was conducted in Meuhedet, Israel’s third-largest state-mandated health maintenance organization (HMO), serving over a million clients from all across the country, which has implemented the “Hybrid Medicine” program as part of its primary care health services. This program allows patients to choose between traditional, face-to-face in-clinic visits and a telemedicine approach via video visits, phone visits, or SFF with the same PCP. In Meuhedet’s HM, instead of a call from whichever PCP is available, the patient schedules a visit or sends a form to a personal physician, who has full access to the patient’s medical file. This ensures not only a personal connection, but consistent attention from the same medical professional. The HMO offers the VV service via a technological platform called *American Well*, which was first implemented in May 2019. Patients can schedule VV and PV appointments, the same way they would in-office visits. This HMO was chosen due to the relatively high number of patients scheduling telemedicine appointments, i.e., they had experience with all visit types under investigation in the current study”.

### Focus groups with patients and physicians

Four focus groups comprised of 24 patients using hybrid primary care service in the Meuhedet Health Services HMO (around 7 in each FG) and four groups comprised of 33 PCPs participated in the focus groups with around 8 in each group.”

Convenience sampling was used for patients and PCPs. The number of focus groups was determined by the “data saturation” principle; that is, data are analyzed until additional data gleaned from the interviews no longer contribute to the understanding of the topic under study [26]. The size of the focus groups encouraged the expression of a diversity of opinions, without overburdening the group with too many participants [24]. Physicians and patients were not compensated for their time. The meetings were held in parallel and independently Experienced moderators – facilitated the group discussions. The researchers contacted Meuhedet patients by Facebook and invited them to participate in the focus groups. Each focus group was conducted in Hebrew and lasted 40–60 min. As all focus groups were conducted during the COVID-19 pandemic period, which required social distancing, the interviews took place remotely via Zoom. The video Zoom file was deleted immediately after the group discussion; only the audio record was saved, which was later transcribed verbatim. Prior to the Focus group, the moderators introduced themselves to the participants, and described the study aims. At the beginning of the interview, participants were informed that only the Audio file of the meeting would be saved and used in the research, and were asked for permission to record the meeting once again.

### The interview guides and think aloud technique

The interview guides were developed by a multidisciplinary team that included two behavioral science researchers and two policy makers, who are physicians specializing in family medicine. The interview guides are semi-structured in nature, thereby covering the main pre-determined themes and attributes, and allowing the interviewers to follow the interviewees’ narrative lines. Open questions were used to obtain unprejudiced information, followed by additional cues to adhere to the interview schedule and aims. In the patients’ Focus groups, the objective was to deepen our understanding of those attributes that influence their choice preferences from among four types of meetings with their PCP: face-to-face, in-clinic visits; and three telemedicine options: VV, PV, or SFF, using the Think Aloud technique. Physicians’ Focus groups included two questions to understand which attributes physicians perceive as important to patients regarding the use of different telemedicine channels. Other questions in the physicians’ focus group were beyond the scope of this study. The first patients’ and physicians’ focus group was transcribed and analyzed immediately afterwards, to confirm that the interviewers captured all useable data.

*Think Aloud* is a research method used to study cognition, and is considered the optimal method to capture thought processes [27]. “Think Aloud” occurs when individuals verbalize their thoughts while performing a task. This approach was adopted in a current study among the patient population to better understand their decision-making process, and the attributes that influence patients’ decisions when choosing which visit type they prefer: face-to-face in-clinic visits vs. telemedicine visits. While the Think Aloud technique may not decrease responder burden, it may enable more accurate attribute identification and reconfiguration. Think Aloud data can be obtained according to two methods: concurrent and retrospective [28]. In the current study, patients were first asked to perform a “warm-up” exercise by doing a Think Aloud task in order to practice and better understand the technique.

They were asked to count the number of windows in their home, thinking aloud as they went through their home’ rooms [29]. Then, patients were asked to answer questions by verbalizing their thoughts *concurrently*, by sharing their impressions and opinions. Patients’ and physicians’ interview question samples and full interview guides are provided in the Supplementary Materials section.

### Data analysis

Thematic analysis [30] was conducted to analyze the data collected in patients’ and physicians’ interviews. Eight attributes with relevant levels were identified from patients’ data, five of which also emerged in the physicians’ focus groups. These attributes will be presented in the Results section. A category tree was built, which included 8 mapped themed attributes. The themes were ascribed a, b, and c signs, reflecting how many times the theme was mentioned, and how strongly each theme was. The transcription and analyzation was done in Hebrew, quotes were later translated into English.

### Attribute ranking exercise

After analyzing the data collected from the patients’ focus groups, a new convenience sample comprised of 48 patients was formed. Participants were asked to rank attributes to ensure that the four most important attributes would be selected for the future DCE. This ranking exercise was built using the Qualtrics web application. Patients were asked to choose the *“four most important attributes when choosing from among four health service channels: Face to Face visit, video visit, phone visit, and Store & Forward written form*” out of all the attributes previously identified. Ranking frequencies of those pre-defined attributes were calculated using Excel software. The top four ranked attributes in each stakeholder’s group were selected for a future DCE experiment.

## Results

### Patient and physician characteristics in focus groups

The focus group participants varied in age, sex, and geographic area. Additional specific variables included their spoken language. Table 1 presents the characteristics of the patients and PCPs who participated in the focus groups, respectively.

**Table 1:** First phase: Focus Groups – Patient and PCP characteristics

Characteristic	*N* Patients [*n* = 24]	PCPs [*n* = 33]
Sex, Female	13	12
Mean Age (SD)	40	50
Geographic area		
Center of Israel	18 (75%)	17 (51%)
Periphery of Israel	6 (25%)	16 (48%)
Specialty		
Pediatrics		15 (45%)
Family Medicine		18 (55%)
Primary Language	Hebrew (100%)	Hebrew (94%) Arabic (6%)
PCP Employment Type		
Self-employed		12 (36%)
Employed by HMO		21 (64%)
SFF with PCP, user	24 (100%)	
PV, user	20 (83%)	
VV, user	17 (70%)	

*VV* Video Visit, *PV* Phone Visit, *SFF* Store and Forward Form, *PCP* Primary Care Physician

### Focus group results

Eight attributes described by two levels each were identified in the patients’ focus groups presented above. Three of them are ascribed with an “a” sign, reflecting their importance and frequency in the focus groups: Waiting time until appointment (day/three days); Queuing time before consultation (5 and 30 min); The severity of the problem (small problem/big problem); and Risk of infection (severe risk/mild risk). Four remaining attributes, perceived as being less important were: Arrival time to the clinic (long time/short time); Relationship with the physician (deep familiarity/superficial familiarity); Flexibility of the PCP’s reception hours (flexible/inflexible); Patient type (the visit is for me/the visit is for my child). *Waiting time until appointment* was an important attribute which the patients discussed extensively in the focus groups. Patients said they preferred the closest appointment (time-wise) when choosing one out of the four channels. Patients considered FV and VV more suitable for *big medical problems* and PV and SFF for mild*, small medical problems*. Patients felt more comfortable conducting all virtual types of medical service when their PCP *“knew them well”.* When PCP reception hours were more flexible, patients had a higher preference for Face to Face and video visits. In contrast, when the hours were less flexible, it seems that phone visits and SFF were the preferred channels (Table 2).

**Table 2:** Quotations from Patients and PCPs using Hybrid Medicine, which contributed to themes for attribute and level development

No.	Sign	Attribute	Description	Identified Levels	Quotations from the Patient groups	Quotations from the PCP groups
1	A	Time until appointment	Typical waiting time until the appointment with a non-acute problem	1 day/ 2 days	*“Why leave the house if you can get the same service and save the time spent waiting for the appointment?” (1) “I had a case with my daughter .... there was no appointment available, everything was booked several days ahead. Then I made a digital appointment for the same day. There were two available appointments: one in the afternoon and one in the evening. So, I made a digital appointment [video] for 7 pm that same day”. (1)*	*“There’s no doubt that this allows patients to see the doctor faster.” (1)*
2	A	Queuing time before consultation	Typical queueing time before consultation	5 min/ 30 min	*‘Usually [the consideration] is the queuing time. When I make an in-clinic appointment, I feel like I’m waiting for the PCP, [but when I make a virtual appointment, it’s as if] the PCP is waiting for me” (2). Queuing time before consultation played a very significant role here.” (1)*	
3	A	The severity of the problem	Perceived severity of the patient’s non-urgent medical problem	Big problem/ Small problem	*“If it’s all sorts of small things, let’s say related to the skin - skin diseases, some kind of fungal infection, I prefer to see my PCP digitally. [But], if I had a concern about skin cancer, I might have gone to the PCP to see him [frontally]” (1) “If I don’t need anything serious, then why should I waste my time going to the PCP [physically]?” (2)*	*“If there is a small problem, patients [tend] to prefer a phone visit…and prefer a video [call] if it’s more of a big problem.” (3)*
4	A-B	Risk of infection	The degree of danger of being infected (not necessarily by COVID-19)	Severe risk/ Mild risk	*“…And the [idea of the risk of] infection, which hangs over the clinics nowadays. It’s impossible to know what you might catch – that’s definitely one of the considerations.” (1) Whenever I come to the clinic, there are lots of people there - coughing, sneezing. Why do I need to get infected?”(3)*	*“Now with COVID-19…I’m from an area that’s highly infected, so people try not to come if they can. So, I think [telemedicine] is a good tool.”(4)*
5	B	Arrival time	Time needed to arrive to the clinic and find parking	Long time/ Short Time	*“No need to waste my time - either on the road or in line. I come in[to the room] a minute before [the video/phone call], [it’s] very convenient*.” *(1)“If I go to see the PCP [physically], it takes a few hours… but, if I see the PCP* via *a video call, it only takes as long as the conversation itself.” (2)*	*“The patient’s time is also of value in my opinion - no less than ours, There’s no doubt that for every minute we invest, they save half an hour of driving to the clinic and finding parking.” (1)*
6	B	Relationship with PCP	The previous history, depth, and significance of the patient-PCP relationship	Deep/ Superficial	*“Of course, if I know the PCP already, it’s much easier to trust him from a distance.” (2) If it’s a doctor I already know - he knows me, I know him - so…you know, we already have ‘a common language’. Then, maybe it’s easier for me…that’s why I’ve already had a few video visits.” (3)*	*“For patients I know and have treated before – [meaning],I know his background, his diseases…[I feel comfortable] giving him digital service - either* via *video or online form.”(2)*
7	B	Flexibility of hour in the schedule		Flexible / Non-flexible	*“Physical meetings with a PCP, from my personal experience, were always pushed into my schedule - either very early in the morning or late in the afternoon. Hours like ten o’clock, one o’clock or three o’clock, are just not realistic times for me, as someone who works full time. Digitally, [the appointment] can be anytime.” (2) If I need something not during his reception hours, I’ll call him, I’ll ask him, I’ll send him a WhatsApp message and ask if he can talk to me. Then, I’ll say okay…the call is recorded…it’s official.” (3)*	
8	C	Patient type	The patient is in need of medical advice	Adult/ Child	*“If it’s for me, I’ll do it digitally; but if it’s for my child, I will come to the clinic.” (3)*	

Focus group patients mentioned two queuing times: “a long time” and “a short time”. When asked, it seemed that “a short queuing time” was considered 5 min and “a long queuing time” ranged from 30 to 40 min. In order to re-validate, a literature review took place. We reviewed previously published research on waiting times in Israeli HMOs, queuing-times related to Israeli HMO reports, and Ministry of Health control reports on queuing times in HMOs. In the end, 5- and 30-min queuing times were validated and set as final.

The physician focus groups were part of a larger study, whose aim was to examine the promoters and barriers of Hybrid Medicine implementation and effectiveness on primary care health systems. It was important for us to explore physicians’ perceptions regarding patient’s preferences and usage. The PCP focus groups revealed five attributes of the eight identified in the patient focus groups: time before the visit, arrival time, risk of infection, relationship with PCP, and the severity of the medical problem. For the physicians, it was also clear that availability was a main factor for the patients. “*The patient’s time is also of value, in my opinion – no less than ours*”; *“There’s no doubt that this allows patients to see the doctor faster”*. The physician’s relationship and familiarity with the patient and his/her history was crucial for them. *“For patients I know and have treated before – [meaning], I know his background, his diseases…[I feel comfortable] giving him digital service - either via video or online form”.* As the focus groups were conducted during a lockdown, the issue of becoming infected was raised often: *“Now with COVID-19…I’m from an area that’s highly infected, so people try not to come if they can. So, I think [telemedicine] is a good tool”.*

### Ranking attributes results

In a convenience sample, 48 patients were asked to rank attributes to ensure that the four most important attributes would be selected for future DCE. The participants’ background variables varied in terms of age, sex, geographic area, and additional specific variables (Table 3).

**Table 3:** Attribute-rankig exercise: Patient characteristics

Characteristic	Patients [*n* = 48]
Sex, Female	37 (77.08%)
Mean age, years	41.7
Marital status, Married	34 (70%)
Mean Level of Education, in years,	15
Family income status, n (%)	
< NIS15,000	8 (16%)
Average NIS15,000	5 (10%)
> NIS15,000	34 (70%)
Chronic Disease, No	38 (79.1%)
In-office follow-up visits in the past year	20 (41.6%)

*NIS* New Israeli Shekel

**Fig. 1:**
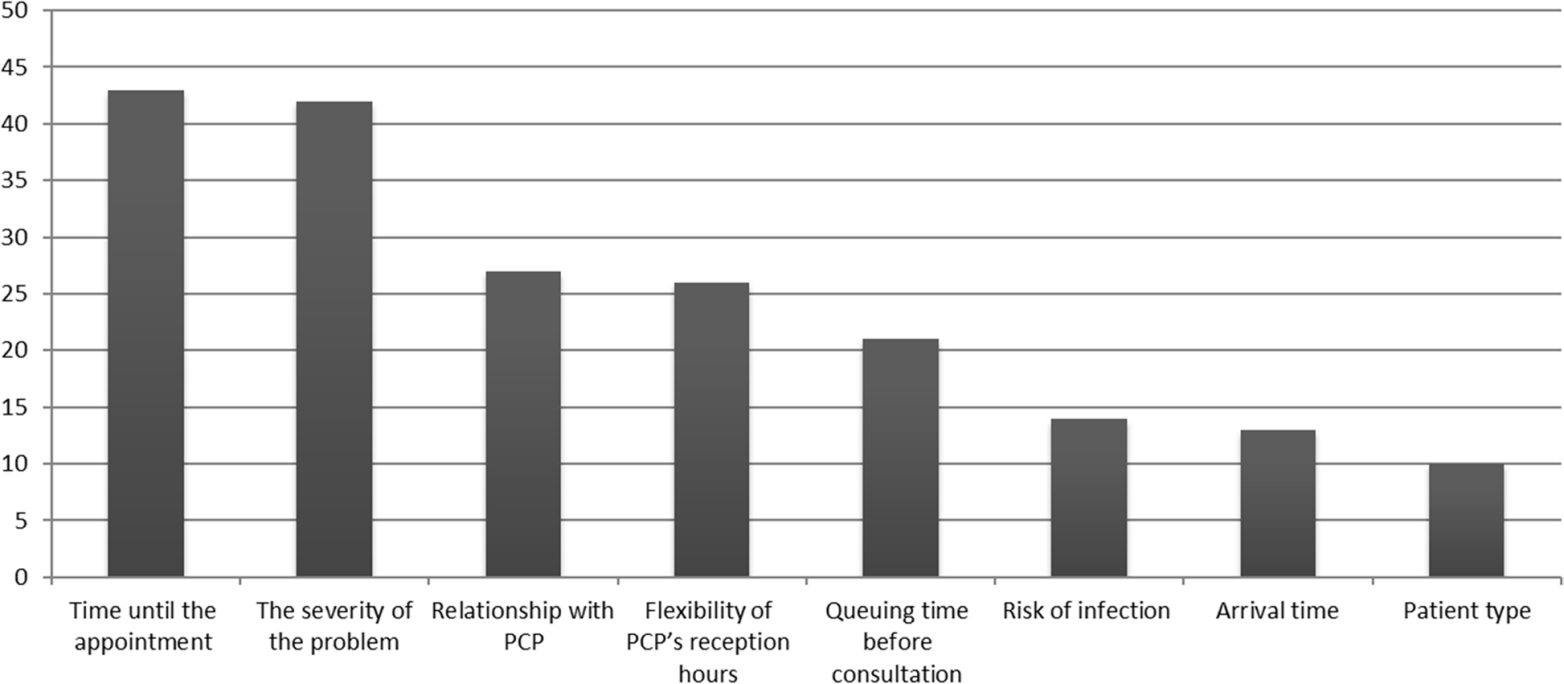
Patients’ selection of top 4 attributes (ranked in order of importance)

Four out of eight attributes were selected by this group of patients as the most important attributes (Fig. 1). First, *time to next available appointment* was found to be an important consideration. The second attribute was *the severity of the medical problem*. Another attribute which emerged as being essential for these participants was the *personal relationship with PCP*. Finally, *flexibility of PCP’s reception hours* was also selected as one of the most relevant attributes.

The study identified eight attributes relevant to medical services selection. In the next quantitative DCE phase, four of these attributes were elicited. The researchers’ choice to limit the upcoming quantitative DCE experiment to four attributes, in addition to the labeled variables (FV, VV, PV, and SFF), was based on the aim to regulate the cognitive burden of survey respondents [31]).

## Discussion

The traditional DCE is one of the most established choice-based formats used to elicit stated preferences regarding health [31]. This study presents a qualitative analysis using DCE attributes and levels, and the Think Aloud approach. It adds new knowledge about user-patients’ preferences toward four different options for conducting a consultation with a PCP, who works in a multi-channel hybrid practice. The COVID-19 period enhanced the adoption of these technologies, and it is likely that there has been a shift in patients’ preferences following their remote consultation experiences with their PCP during this period. Thus, our findings contribute to a better understanding of patients’ preferences during the COVID-19 period.

The most important attribute identified in the study was *Waiting time to next available appointment*. On 2019, Israel Ministry of Health has instructed HMOs to set precise targets for maximum waiting times for specialists, and to publish these on their websites. This attribute has been extensively discussed in the literature and was found to be significant to patients’ preferences when referring to meeting a family physician in clinic or via a video visit [16, 17, 32]. Furthermore, this attribute was also expressed by the physicians in our focus groups, identifying with the attribute. *The severity of the problem* was the second most important attribute. In regard to “administrative matters” or a “small medical problem”, patients choose to use the PV and SFF; however, when the medical problem was perceived as more serious, they preferred to contact the physician face to Face or via video. The ability to see the face of a physician, whether from a distance or up close was an important factor related to the severity.

The *Patient-PCP relationship,* and whether or not they were familiar with one another played an important role in the choice of preferences – for both patients and a very important factor for physicians. When patients were familiar with their physician, they tended to choose VV and PV more frequently. Hence, the level of acquaintance and intimacy impacted their decision, which is also supported in the telemedicine preference literature [16, 33]. In such a case, patients and physicians felt more comfortable to use a PV, even for a long call. It seems that in the decision-making algorithm, when the problem was more severe and when their PCP knew them well, the patients’ preferred choice was a video visit, but this issue requires further examination in the quantitative phase of the DCE in order to understand it within a whole estimate model, showing tradeoffs between all considered attributes. The fourth attribute noted by patients as being of importance was *flexibility of PCP’s reception hours*. In patients’ focus groups, “flexible hours” referred to “how many hours a week the PCP is available” and whether “the PCP receives patients in the evening”. If the PCP kept inflexible reception hours, the patients’ tendency to choose PV and SFF was greater. This is a significant finding related to patients’ preferences to use telemedicine, since more significant and high-quality visits – according to the values ​​of family medicine – are considered to allow for a higher degree of intimacy during the visit [19, 34]. This is possible more so in Face to Face and video visits, and less possible via phone visits and written requests. Therefore, policymakers should consider the issue of PCP reception hours to allow patients to choose an intimate, and more significant visit.

In a previous DCE study, conducted in Israel prior to COVID-19 about non-user/patient and PCP preferences [16], one of the main attributes selected by patients and PCPs was Quality of consultation defined by *the PCP’s attention to the patients during the remote video visit*. However, this attribute was not expressed as being important by users: either patients or PCPs in the current study physicians that used the video-visit defined the usage as “intimate” and said: “I use the camera to enter their [the patients’] home, kitchen, refrigerator, and bedroom, and in fact make an assessment of their mental state, as well as an assessment of their economic situation”. Surprisingly, *risk of infection* was raised in the physicians’ focus group and as an attribute of choice both by patients and PCPs, but it was ranked in sixth place among the eight attributes. The significance of this finding may suggest that despite the COVID-19 pandemic other factors are still more significant when it comes to making choices and expressing preferences on a routine basis.

A methodological contribution of the study lies in both a detailed description of the attribute identification process, including the number of steps and tools: (1) a literature review, (2) focus groups with targeted populations using Think Aloud technique interview guides, and (3) a ranking exercise. The rationale behind using the Think Aloud technique in DCE studies was discussed previously in the literature, but studies that describe the experience with this technique are limited [35–37]. The lessons learned from using the technique are described below. When the goal in qualitative DCE is to select attributes and levels for the choice between a relatively large number of options, using the Think Aloud technique is recommended and perhaps even necessary. The citations from patients’ focus groups described in Table 3 depict a clear description of how decisions on attribute and level selections were processed, following “expressing the thoughts aloud”, and are the rationale behind the trade-off made. When patients choose from among several options, the decision-making algorithm is complex, and thinking aloud enables the surfacing and expression of attributes of which the patients themselves may not be sufficiently aware. Employing this technique was challenging. Despite the request of focus group moderators to “put their thoughts into words”, patients tended to ignored this request and, at first, gave “regular” answers about attributes without sharing their thought process. Only when interview moderators requested a second or third time to express their thoughts aloud, did the patients do so. Older focus group patients seemed to find it harder to remember to express their thoughts aloud compared to the younger patients. Therefore, when researchers make the decision to use the Think Aloud technique, the duration of the focus groups needs to be longer than the usual hour/hour-and-a-half. This may help more patient-participants understand how to express their thoughts aloud. Another methodological insight addresses the importance of identifying attributes and levels within the population where a quantitative phase of the DCE is going to take place (the patient population in our case.). Physicians interviewed in the current study indicated only two out of the four attributes which patients said were important. Appointment availability waiting time and flexibility of PCP’s reception hours were seemingly unimportant to physicians and were only raised in the patients’ focus groups. This supports the recommendation [18, 38] to identify attributes for DCE studies within a targeted population. In studies that examine patients’ preferences, in order to better understand patient attributes, it is not enough to conduct a literature review and consult physicians; the patients themselves need to be the main population identifying what is important to them.

Our study has a number of limitations. Since the focus groups were conducted in Hebrew, Arabic-only-speaking Israeli patients could not participate in the study; thus, their preferences remain unknown. In addition, the current study included only one health organization; therefore, the present results’ generalizability to other settings requires further examination. Another limitation refers to the generality of the results. Since the study was conducted in the Israeli health system, in which every citizen has National Health Insurance including telemedicine services, the willingness to pay attribute was not raised in the focus groups. In other health systems, where a rate is charged for different telemedicine services, this variable will surely be a relevant attribute when it comes to patients’ choice. ​​Another limitation is the representativeness of the ranking exercise done based upon information of the focus groups though qualitative research does not require a representative sample. We are continuing our research these days with a large patient survey. Lastly, the study was conducted during the COVID-19 pandemic; hence, the conclusions may relate only to this period.

## Conclusions

This paper describes the attribute and levels identification process prior to quantitative DCEs, based on qualitative work and a targeted population using the Think Aloud technique. The usage of this technique has the potential to provide unique benefits in qualitative research on DCEs; and hence, should receive more attention in future DCE studies. This study’s theoretical contribution is post-COVID-19 patients’ preferences in a cost-free Hybrid Medicine patient care This provides a foundation to assess the rigors of this stage and provide additional evidence to the limited existing literature on attributes development for DCE patient preferences.

## Supplementary Information

**Additional file 1.**

**Additional file 2.**

## Data Availability

The datasets during and/or analyzed during the current study available from the corresponding author on reasonable request.

## Abbreviations

COREQ: Consolidated Criteria for Reporting Qualitative Research
DCE: Discrete Choice Experiment
FV: Face-to-face, traditional in-clinic consultation
HM: Hybrid Medicine
HMO: Health Maintenance Organization
PCP: Primary Care Physicians
PV: Phone Visits
SFF: Store-and-Forward written digital form
VV: Video Visit
