# Supplementary material for: Patients’ preferences for telemedicine versus in-clinic consultation in primary care during the COVID-19 pandemic
Source: BMC Prim Care. 2022 Feb 22;23:33. doi: 10.1186/s12875-022-01640-y (PMC8862698; doi:10.1186/s12875-022-01640-y)
Supplement: Supplementary file 1 — Additional file 1. [file 12875_2022_1640_MOESM1_ESM.docx]

**Interview Guide for Patients**

**Interview Introduction**

Thank you for agreeing to participate in this focus group, which we are conducting as part of a study on patients' preferences when consulting with a family physician and/or pediatrician. Your participation in the study may make a significant contribution in furthering our understanding of the subject. The interview will last about 30-60 minutes.

Everything you say during the interview is confidential and will be used solely for the purposes of the research. With your consent, I will record the group interview, which will later be transcribed. We will not save the interview’s video file ; only the audio Zoom file will be saved. From this point on and throughout the research, we are committed to maintaining confidentiality and will not disclose any information that could identify you or personally link you to what was said during the interview. If you are asked questions that you do not want to answer, or asked about topics that you would prefer not to address, we will respect your wishes. You can stop participating in the interview at any time.

**Questions**

You can currently receive medical services from your family physician (GP) in four ways: via a face-to-face in-clinic visit; a video visit; a phone call visit; or a Store &Forward form. Which of these options do you prefer most? Which do you use when you want to engage your physician’s services?

**Thinking Aloud** –

- In the upcoming series of questions, we will ask you to answer in a way that allows us to understand your decision-making process. It is important to speak freely and express your thoughts and opinions when answering a question. In order to practice this way of answering a question, we will first ask you a question which is not related to the study topic: How many windows does your home have? Use the Think Aloud method: Go through each room in your home and count aloud the number of windows as you go. Thank you (short feedback on answering the question).
- Think Aloud Question 1: Now let’s discuss visits to your physician. Imagine you are interested in contacting a physician regarding a non-urgent medical problem. What type of consultation would you prefer : face-to-face, in-clinic visits or one out of three types of telemedicine visits (video visits, phone visits, or Store & Forward form. Please share your decision-making process regarding your consultation preferences.
- Express your preferences using a “for and against each scenario” approach. In your opinion, what are the benefits of each option?
- From among the three telemedicine options, which option do you prefer most? video visits / phone visits / Store and Forward forms
- How would you prefer to hold this type of telemedicine consultation? from a home computer/phone / from a work computer/phone / from my smartphone
- What is important to you in this decision making?
- Think Aloud Question 2: (Here the interviewer will formulate a new question about the characteristics and levels that emerged from the focus group interviews. For example: “What would you prefer? To visit the doctor within five days, to wait two hours for a consultation itself and to see a doctor face to face or To visit the doctor within one day, to wait half an hour for a consultation itself and to see a doctor via video?
- Think Aloud Question 3: (Here, once again, the interviewer will formulate a new question about the characteristics and levels that emerged from the focus group interviews.
- What are the four most important considerations among those raised in the discussion? (Here the interviewer will list different attributes that came up in the discussion.)

*Three more question were asked, which are beyond the scope of this article.
