# Supplementary material for: Patients’ preferences for telemedicine versus in-clinic consultation in primary care during the COVID-19 pandemic
Source: BMC Prim Care. 2022 Feb 22;23:33. doi: 10.1186/s12875-022-01640-y (PMC8862698; doi:10.1186/s12875-022-01640-y)
Supplement: Supplementary file 2 — Additional file 2. [file 12875_2022_1640_MOESM2_ESM.docx]

**PCP Interview Guide**

**Interview Introduction**

Thank you for agreeing to participate in this focus group, which we are conducting as part of a study on the preferences of patients and primary care physicians regarding Hybrid Practice. This is a joint research project conducted by the Meuhedet.org Health Services and the University of Haifa. The term “Hybrid Practice” refers to a combination of four types of consultation options for meeting a physician: traditional, frontal in-clinic visits; video visits; phone visits; and online inquiries (Store & Forward form).

Your participation in the study can make a significant contribution and further our understanding of the subject. The interview will last about one hour.

Everything you say in the interview will remain confidential and will be used solely for the purposes of the research. With your consent, I will record the group interview, which will then be transcribed. The video file will not be saved in our files – it will be deleted immediately after the interview. From this point on and throughout the research, we are committed to maintaining confidentiality and will not disclose any information that could identify you or link you personally to what was said during the interview.

If you are asked questions that you do not want to answer or if we bring up topics that you would prefer not to address - we will respect your wishes. You can stop participating in the interview at any stage. In accordance with Ethics Committee approval, we can only save the audio file of the interview, but not the video file.

**Questions**

**What are the benefits and challenges of Hybrid Practice?**

Patients’ preferences regarding how to contact you:

- Based on what your patients share with you, and based on your personal understanding, how do your patients decide which telemedicine channel to use when they want to meet with you? From among the following four options, which would they choose? Face-to-face, in-clinic visits; video visits; phone visits; or Store & Forward form.
- Now think about your “user”-patients – the patients who use all four of the above-mentioned telemedicine channels. How do you think they decide from among the four different options?

*Here, six other questions were asked, which are beyond the scope of this article. The questions dealt with physicians’ agenda regarding Hybrid Practice, their experiences with each of telemedicine channels, burnout, aspects of hybrid practice, and more.
